# A synbiotic combination of mixed probiotics and oligofructose restores intestinal microbiota disturbance in DSS-induced colitis in mice

**DOI:** 10.3389/fmicb.2025.1582155

**Published:** 2025-07-24

**Authors:** Weiwei Ma, Lian Lian, Lidong Guo, Yanan Wu, Lili Huang

**Affiliations:** College of Pharmacy, Heilongjiang University of Chinese Medicine, Harbin, China

**Keywords:** *Bifidobacterium*, *Lactobacillus*, oligofructose, colitis, short-chain fatty acids, gut microbiota

## Abstract

Both probiotics and prebiotics can regulate gut microbiota and produce metabolites that are beneficial to the host, showing potential therapeutic prospects for ulcerative colitis (UC). This study aimed to investigate the therapeutic effects and mechanisms of low-, medium-, and high-dose probiotic mixtures (*Bifidobacterium animalis* subsp. *lactis* XLTG11: *Lactobacillus paracasei* Glory LP16: *Lactiplantibacillus plantarum* CCFM8661) combined with oligofructose (FOS) on DSS-induced colitis mice. Male BALB/c mice were induced with 2.5% DSS for colitis and then treated with low-, medium-, and high-dose mixed probiotics and oligofructose synbiotics (6 × 10^6^ CFU probiotics + 1.5 mg FOS synbiotics/day, 6 × 10^7^ CFU probiotics + 1.5 mg FOS synbiotics/day, and 6 × 10^8^ CFU probiotics + 1.5 mg FOS synbiotics/day) for 1 week. Colonic inflammation was evaluated by body weight, diarrhea index, immune organ index and colon length; the protective effect of synbiotics on the intestinal barrier was evaluated by HE staining, ELISA and RT-qPCR to detect the degree of intestinal tissue damage, inflammatory cytokines, intestinal permeability and tight junction proteins. In addition, the intestinal microbiota and short-chain fatty acids (SCFAs) were estimated by metagenomics and LC-MS, respectively, to analyze the mechanism of action of synbiotics in alleviating colitis. The results showed that the combination of mixed probiotics and oligofructose could significantly alleviate the symptoms of colitis in mice, improve the weight loss symptoms of DSS mice, reduce the diarrhea index, restore the colon length, reduce tissue pathological damage, and improve the integrity of the intestinal barrier. In addition, through metagenomic data analysis, mixed probiotics combined with oligofructose can enhance the diversity and richness of gut microbiota, increase the abundance of beneficial bacteria *Bifidobacterium* and *Lactobacillus*, promote the production of SCFAs, and enhance intestinal barrier function. It is worth noting that the therapeutic effect of synbiotics depends on the dose of mixed probiotics. This study explored the alleviating effect of synbiotics containing mixed probiotics and oligofructose at different doses on UC, and provided a theoretical basis for guiding the development and use of synbiotic preparations that regulate the homeostasis of gut microbiota.

## 1 Introduction

Inflammatory bowel disease (IBD) refers to a collection of illnesses that are typified by chronic and recurrent inflammation of the gastrointestinal tract. The two main subtypes of IBD are ulcerative colitis (UC) and Crohn’s disease (CD) (Dowdell and Colgan, 2021; Kobayashi et al., 2020). Incidence and prevalence of UC are rising globally. UC is characterized by intestinal mucosal damage, intestinal microbiota imbalance and metabolic disorders, and inflammation begins in the rectum and extends to the proximal colon (Kobayashi et al., 2020). The exact mechanism leading to the development of UC is still unclear, but studies have determined that an imbalance in the intestinal microbiota is at the core of UC pathogenesis (Hirano et al., 2018; Matsuoka and Kanai, 2015).

Trillions of bacteria, viruses, fungi, unicellular animals, and archaea make up the human intestinal microbiota, which is primarily found on the surface of the colon and distal ileum (Lu et al., 2022). Compared with healthy people, the intestinal microbiota of UC patients shows common microbial characteristics, namely decreased microbial diversity, reduced Firmicutes, and increased Proteobacteria (Gevers et al., 2014). The gut microbiota regulates the activation of the innate immune system through intestinal metabolites and plays a crucial role in the pathogenesis of UC (Lu et al., 2022). Balanced intestinal microorganisms can improve the host’s nutrient supply by producing beneficial bile acids (BAs), short-chain fatty acids (SCFAs), and vitamins. It plays a vital role in maintaining human health by preventing pathogen colonization and maintaining normal mucosal immunity (Lavelle and Sokol, 2020). Important intestinal metabolites known as SCFAs affect intestinal microbiota, control intestinal pH, and are crucial for intestinal health including epithelial growth, mucus secretion, and immune regulation. Studies have shown that the levels of SCFAs in the feces of UC patients are reduced to varying degrees (Bjerrum et al., 2015; Vernia et al., 1988). Acetate, propionate, and butyrate are the main SCFAs produced by intestinal bacteria, which help maintain the barrier function of the mucosal surface epithelium and regulate the host’s intestinal immune system (Mann et al., 2024; Xu et al., 2024). The gut microbiota and its metabolites, SCFA, are therefore thought to be key drivers for reducing the inflammatory response linked to UC.

“Synbiotics” are probiotics and prebiotics together. Probiotics break down and metabolize prebiotics into monosaccharides or disaccharides, which facilitate colonization and proliferation. The growth of probiotics is positively impacted by prebiotics (Jadhav et al., 2023; Roy and Dhaneshwar, 2023). The synergistic effect of the two is more significant than that of either component alone (Jiang et al., 2024). Dietary supplementation with synbiotics is becoming a promising method for treating UC and has potential application value. For example, synbiotics containing *Bifidobacterium longum, Lactobacillus acidophilus* and seabuckthorn polysaccharides can alleviate the symptoms of DSS-induced colon inflammation. Studies have shown that dietary supplementation with synbiotics is more beneficial for relieving colitis than the use of probiotics and seabuckthorn polysaccharides alone (Yuan et al., 2024). Oligosaccharides are one of the three major prebiotics and are widely used. They can regulate intestinal microbiota, increase lactic acid bacteria and bifidobacteria, promote the production of healthy SCFA, and regulate the intestinal immune barrier (Costa et al., 2022). Therefore, using oligofructose as the main component of synbiotics has great potential to alleviate intestinal inflammation.

The benefits of lactic acid bacteria or bifidobacteria in combination with prebiotics on the relief of colitis in mice are now the subject of numerous investigations. However, there are few studies on the protective effects and mechanisms of different doses of probiotics combined with prebiotics on colitis in mice. In order to determine whether increasing the dose of mixed probiotics can enhance the potential of synbiotics in treating UC, this study selected low, medium and high doses of mixed probiotics (*Bifidobacterium animalis* subsp. *lactis* XLTG11: *Lactobacillus paracasei* Glory LP16: *Lactiplantibacillus plantarum* CCFM8661) combined with oligofructose to explore its protective effects and mechanisms on DSS-induced colitis mice from the aspects of intestinal inflammation, histopathology, intestinal barrier, and intestinal microecology. This study will contribute to the subsequent development of synbiotic products.

## 2 Materials and methods

### 2.1 Preparation of probiotic strains

Bacterial strains, including *Bifidobacterium animalis* subsp. *lactis* XLTG11 and *Lactobacillus paracasei* Glory LP16 were purchased from Jinhua Yinhe Biotechnology Co., Ltd. *Lactiplantibacillus plantarum* CCFM8661 was purchased from Micro-Bio (Suzhou) Co., Ltd.

### 2.2 Colitis model induction and experimental design

The Animal Experimental Ethics and Safety Committee of Heilongjiang University of Chinese Medicine (No. 2024042614; Harbin, China) approved all animal treatments and experiments, which were carried out strictly in compliance with the National Research Council’s Guide for the Care and Use of Laboratory Animals. We bought 7-week-old male BALB/c mice from SPF Biotechnology Co., Ltd. in Beijing, China. With a temperature of 22°C ± 1°C, a humidity of 55% ± 5%, and a 12-h light/dark cycle, the mice were kept in a controlled environment where they were free to eat and drink. Following a week of adaption, 60 mice were split into five groups at random (*n* = 12 per group): low-dose group (M-L), medium-dose group (M-M), high-dose group (M-H), normal group (NC), and model group (MC). The mice in the model group (MC), low-dose group (M-L), medium-dose group (M-M), and high-dose group (M-H) were given 2.5% DSS (Solarbio, Beijing, China) solution to create the acute DSS model, while the mice in the normal group (NC) were given free access to water during the first week. In order to assess the protective effects of the probiotics against DSS-induced UC, in the second week, mice in the M-L group, M-M group, and M-H group were gavaged daily with 200 μL of 6 × 10^6^ CFU probiotics + 1.5 mg FOS synbiotics, 200 μL of 6 × 10^7^ CFU probiotics + 1.5 mg FOS synbiotics, and 200 μL of 6 × 10^8^ CFU probiotics + 1.5 mg FOS synbiotics, respectively. Mice in each group had their body weight and fecal morphology measured daily during the trial. On day 15, killing the mice and collecting their blood through cardiac puncture; removing the colon and measuring its length; flushing the colon with PBS and either fixing it in 4% paraformaldehyde or rapidly freezing it for subsequent analysis; and collecting and storing the contents of the colon at −80°C until analysis.

### 2.3 Hematoxylin and eosin staining

The tissues of the mouse colon were embedded in paraffin, treated with 4% paraformaldehyde, and then sliced into slides that were 4 μm thick. For histological examination, colon tissues were stained with hematoxylin and eosin (H&E). An electron microscope was used to take pictures of the colon tissue.

### 2.4 Enzyme-linked immunosorbent assay

As directed by the appropriate ELISA kits (Bio-Rad, Wuhan), the levels of LPS, DAO, and D-LA in serum and IL-1β, IL-6, and IL-10 in colon tissue were measured and examined using a microplate reader.

### 2.5 Determination of cytokine expression in colon tissue by real-time PCR

The Trizol technique was used to extract total RNA from 50∼100 mg of colon tissue. A reverse transcription kit was used to reverse-transcribe complementary DNA, and SYBR Green was used to conduct a real-time quantitative polymerase chain reaction (RT-qPCR) on CFX96 (Bio-Rad) The PCR program was divided into the first step of preheating (95°C for 30 s); the second step was a two-step amplification method with 40 cycles (95°C for 10 s, 60°C for 30 s); and the third step was melting curve analysis (95°C for 10 s, 65°C for 60 s, 97°C for 1 s). Table 1 displays the primer sequences (the internal reference gene was GAPDH). The 2^–ΔΔ^*^CT^* technique was used to determine the relative expression of mRNA.

**TABLE 1:** Primer sequences used for RT-qPCR.

Genes	Forward (5′–3′)	Reverse (5′–3′)
Occludin	TTGAAAGTCCACCTCCTTA CAGA	CCGGATAAAAAGAGTACGC TGG
Claudin-1	TGCCCCAGTGGAAGATTTACT	CTTTGCGAAACGCAGGACAT
Z0-1	GTTGGTACGGTGCCCTGAA AGA	GCTGACAGGTAGGACAGA CGAT
GAPDH	TGACCTCAACTACATGGTC TACA	CTTCCCATTCTCGGCCTTG

### 2.6 Metagenomic analyses of colon content

High-throughput sequencing was performed according to the method of reference (Zeng et al., 2024). A succinct description The DNeasy Power Soil Kit (Qiagen) was used to extract genomic DNA from colonic contents. Each sample had 1 ng of total DNA amplified, and the DNA concentration was evaluated at A260. In order to create a gene catalog, the libraries were sequenced using the Illumina HiSeq 2000 platform (Illumina, San Diego, California, United States) after the PCR products had been purified and quality checked. To investigate the variations in species and functional composition among samples, abundance cluster analysis, principal component analysis (PCA), Kyoto Encyclopedia of Genes and Genomes (KEGG) homologous mass spectra, and pathway maps were developed based on the species abundance table and function abundance table.

### 2.7 Determinations of SCFAs

Fill a test tube with 180∼200 mg of frozen colonic contents, add 1 mL of distilled water, vortex for 10 min, centrifuge for 25 min at 500 × g and 4°C, and filter the supernatant using a 0.22 μm filter membrane. To the sample, add 50 μL of 30% phosphoric acid solution, 300 μL of acetone, homogenize for 3 min, centrifuge at 13,400 g for 10 min, remove the supernatant, and dilute on the machine as necessary. The specific parameters of LC-MS refer to the method of Li et al. (2024).

### 2.8 Statistical analysis

The experimental data was presented as median or mean ± SD. Duncan’s test and either a one-way or two-way ANOVA were used to assess statistical significance. Or use SPSS (IBM, US version 21.0) to combine Dunn’s post-test with the Scheirer-Ray-Hare test. The statistical significance of the results was configured as ****p < 0.0001; ***p < 0.001; **p < 0.01, and *p < 0.05. ChiPlot (https://www.chiplot.online) was used to draw a histogram of the intestinal microbiota.

## 3 Results

### 3.1 Effect of synbiotics on symptoms and organ index in colitis mice

**FIGURE 1:**
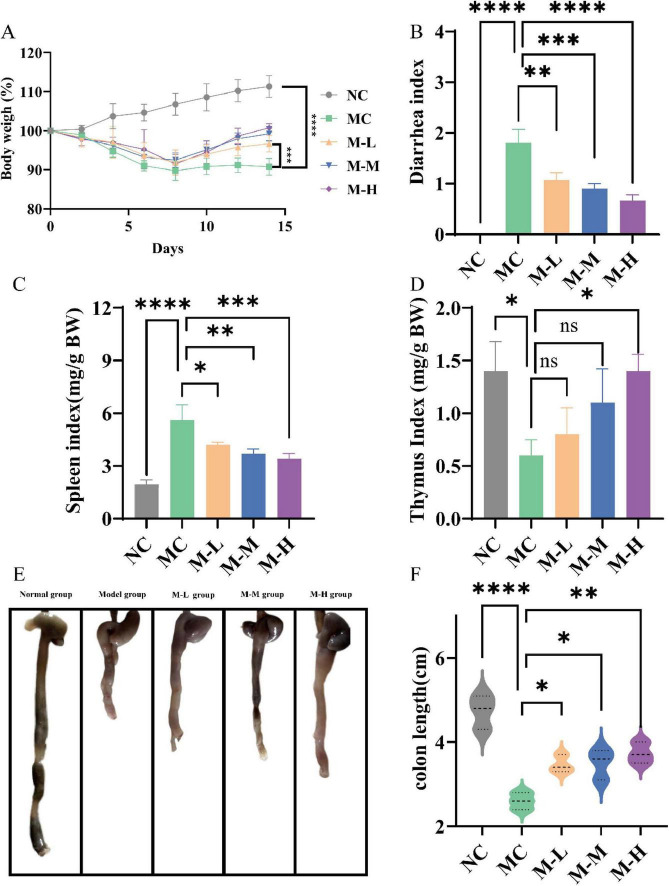
Effect of synbiotics on symptoms and organ index in colitis mice. **(A)** Body weight changes. **(B)** Diarrhea index (DI). **(C)** Spleen index. **(D)** Thymus index. **(E,F)** Colon length (*p < 0.05, **p < 0.01, ***p < 0.001, ****p < 0.0001 and “ns” indicates non-significant, data represent mean ± SE).

Mice were given daily measurements of their body weight and diarrhea score in order to examine the preventive effects of varying dosages of probiotics combined with oligofructose. Weight loss is one of the typical symptoms of DSS-induced colitis mice. As illustrated in Figure 1A, as opposed to the NC group, mice in the MC group and other synbiotic treatment groups showed obvious weight loss symptoms on the third day, and reached the lowest weight on the eighth day. After 7 days of treatment with different doses of probiotics combined with oligofructose, the body weight of mice in all treatment groups was significantly increased compared with the MC group (*p* < 0.001), and M-L group was 5.90% higher than the MC group. In addition, studies have shown that DSS-induced colitis mice are usually accompanied by diarrhea, bloody stools and other symptoms. The severity of diarrhea is usually evaluated by diarrhea index (DI). As shown in Figure 1B, the DI value of mice in the DSS-treated MC group was notably increased (*p* < 0.0001), and severe diarrhea occurred, manifested as wet stools and severe rectal bleeding. The M-L group had remarkably reduced diarrhea scores (*p* < 0.01), and stool morphology was normal.

Increased spleen weight, decreased thymus weight, and shortened colon are indicators for assessing the severity of colitis (Yang et al., 2023). Important immunological organs that produce, store, and release immune cells are the thymus and spleen. As a result, the thymus and spleen indices can somewhat represent the level of inflammation (Lewis et al., 2019). The results are shown in Figures 1C,D. Compared with NC group, the spleen/body weight ratio and thymus/body weight ratio of DSS-induced colitis mice were outstandingly increased (*p* < 0.0001 and *p* < 0.05, respectively). The M-L, M-M and M-H group significantly reversed the DSS-induced effects of the spleen immune organ (*p* < 0.05, *p* < 0.01, *p* < 0.001, respectively); only the M-H group obviously increased the thymus/body weight ratio (*p* < 0.05). The above results indicate that only a high-dose synbiotic of mixed probiotics and oligofructose can simultaneously and effectively resist DSS-induced splenomegaly and thymic suppression, and exhibit a dose-dependent effect. The colon length of mice in the NC group was 4.73 ± 0.40 cm, and the colon length of mice in the MC group treated with DSS was markedly shortened, shrinking to 2.6 ± 0.20 cm, and the colon was atrophic and ulcerated (Figures 1E,F). The intestinal wall turned red, and the feces in the intestine decreased. The synbiotic groups significantly restored the colon length (*p* < 0.05), and the M-H group had the most significant improvement (*p* < 0.01). The above results show that probiotics combined with oligofructose can improve the symptoms of DSS-induced colitis in mice in a dose-dependent manner.

### 3.2 Effects of synbiotics on colonic tissue morphology in colitis mice

**FIGURE 2:**
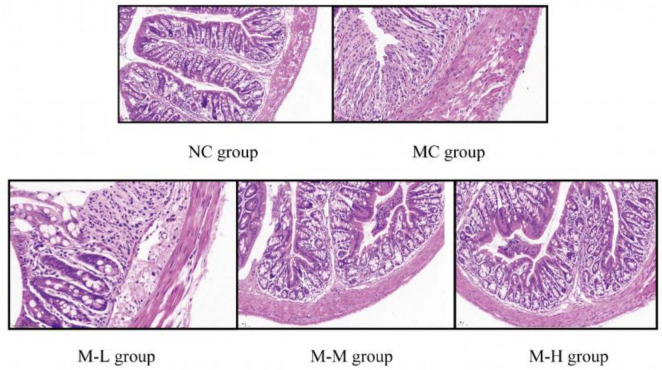
Effect of synbiotics on colon tissue damage in colitis mice.

The colon tissue was stained with HE, and the colon tissue sections were observed under a microscope. The results of histological analysis showed (representative micrographs of each group are shown in Figure 2 that Mice in the NC group had intestinal tissue structure that was intact, goblet cells and crypts were distributed properly, and there was no visible inflammation; the mice in the MC group treated with 2.5% DSS displayed clear colon tissue damage, including the loss of colon epithelial villi and the presence of many infiltrating inflammatory cells (mainly neutrophils and lymphocytes), and the loss of crypts; however, compared with the MC group, the intake of mixed probiotics combined with oligofructose signally reduced the severity of DSS-induced colon tissue damage, and showed a dose-dependent manner, as shown by more severe local damage to the colon tissue of mice in the M-L group, partial necrosis of mucosal epithelial cells, and a slightly stronger degree of inflammatory cell infiltration than the model group; the M-M group’s local damage to the colon tissue was evident, with partially intact tissue structure and a slight amount of inflammatory cell infiltration; the colon tissue epithelial villi in the M-H group were clear, the epithelial cells and goblet cells were arranged neatly, and the crypt structure was intact. As a result, the intervention of mixed probiotics combined with oligofructose treatment group considerably decreased the intestinal tissue damage brought on by DSS, with the M-H group showing the greatest improvement in intestinal tissue damage.

### 3.3 Effects of synbiotics on the production of inflammatory cytokines in colitis mice

Cytokines, as key biomarkers of various inflammatory diseases, play a vital role in determining intestinal health (Neurath, 2024). Proinflammatory factors (including IL-6 and IL-1β) can activate inflammatory cells, enhance systemic or local intestinal inflammatory responses, lead to intestinal immune response disorders, and thus aggravate intestinal tissue lesions. IL-10 is a key anti-inflammatory cytokine that can limit the activation of immune cells of innate immune cell types and inhibit the secretion of proinflammatory cytokines to prevent excessive immune responses and tissue damage (Li et al., 2021).

**FIGURE 3:**
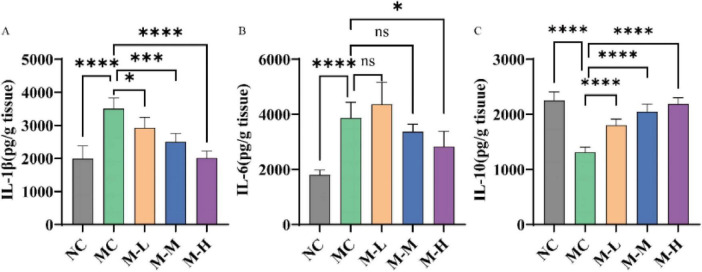
Effect of synbiotics on inflammation in colitis mice. Concentrations of cytokines content, namely interleukin **(A)** IL-1β, **(B)** IL-6, and (C) IL-10 (*p < 0.05, **p < 0.01, ***p < 0.001, ****p < 0.0001 and “ns” indicates non-significant, data represent mean ± SE).

In order to determine the anti-inflammatory effect of the synbiotics of mixed probiotics and oligofructose, the expression levels of proinflammatory factors IL-6, IL-1β and anti-inflammatory factor IL-10 in colon tissue were detected by ELISA in this study. The results are shown in Figure 3. Compared with the NC group, the levels of proinflammatory factors IL-1β and IL-6 in the colon tissue of mice in the DSS-induced MC group were significantly increased, while the level of anti-inflammatory factor IL-10 was markedly decreased (*p* < 0.0001). This result is consistent with the study that observed increased levels of proinflammatory cytokines and decreased levels of IL-10 in patients with UC (Kiernan et al., 2020). After administration of the synbiotic combination of mixed probiotics and oligofructose, the expression of proinflammatory factor IL-1β in the serum of mice in the M-L, M-M and M-H groups was remarkably reduced (*p* < 0.05, *p* < 0.001 and *p* < 0.0001, respectively), and the anti-inflammatory factor IL-10 was significantly increased (*p* < 0.0001). Among them, only the M-H group had a significant decrease in the expression level of serum IL-6 in mice. Although the other treatment groups had a decrease, there was no significant difference from the MC group (*p* < 0.05). In summary, the mixed probiotics combined with oligofructose treatment group can regulate the expression of proinflammatory cytokines and anti-inflammatory factors and prevent the initiation of inflammatory response.

### 3.4 Effects of synbiotics on the intestinal barrier in colitis mice

**FIGURE 4:**
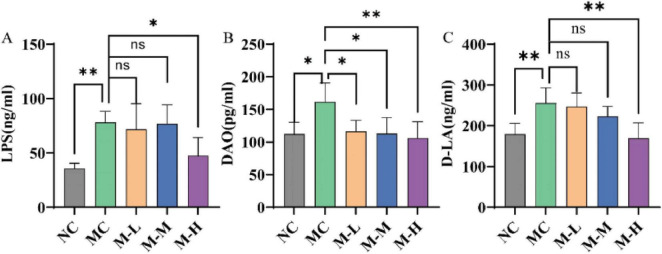
Effect of synbiotics on intestinal barrier in colitis mice. The levels of **(A)** LPS, **(B)** DAO, and **(C)** D-LA in serum (*p < 0.05, **p < 0.01, ***p < 0.001, ****p < 0.0001 and “ns” indicates non-significant, data represent mean ± SE).

One of the pathological characteristics of UC is the destruction of the intestinal barrier. D-lactic acid is a metabolite of intestinal bacteria, and other tissues cannot produce or metabolize D-lactic acid; diamine oxidase (DAO) is an intracellular enzyme that contains deaminated putrescine and histamine, 95% of which are present in intestinal epithelial cells; lipopolysaccharide (LPS) is a lipopolysaccharide in the cell wall of Gram-negative bacteria. Under normal circumstances, these three substances are only present in the intestine and penetrate into the blood under inflammatory conditions (Sun et al., 2015). Therefore, high levels of lipopolysaccharide (LPS), DAO and D-lactic acid in serum indicate that the intestinal barrier is damaged and intestinal permeability is increased. The results of this study are shown in Figure 4. Mice with DSS-induced colitis showed considerably higher serum levels of LPS, DAO, and D-lactic acid (*p* < 0.01, *p* < 0.05, and *p* < 0.01, respectively), but only the M-H group showed notably lower serum levels of LPS and D-LA expression (*p* < 0.05 and *p* < 0.01, respectively).

**FIGURE 5:**
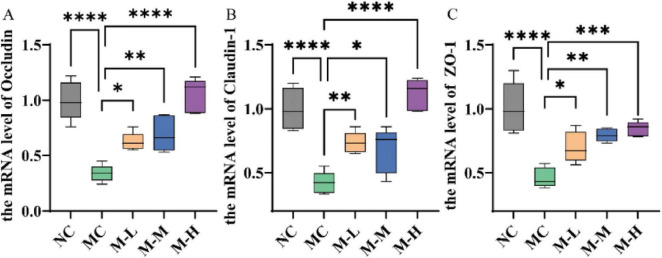
Effect of synbiotics on intestinal barrier in colitis mice. Expression levels of **(A)** occludin, **(B)** claudin-1, and **(C)** ZO-1 mRNA in the colon (*p < 0.05, **p < 0.01, ***p < 0.001, ****p < 0.0001 and “ns” indicates non-significant, data represent mean ± SE).

Additionally, this study also detected the mRNA expression levels of tight junction proteins in colonic tissue to better evaluate the repair effect of mixed probiotics and oligofructose on intestinal barrier damage. Cytoplasmic proteins (ZO-1) and transmembrane proteins (occludin and claudins-1) make up tight junction proteins. They have multiple functions such as regulating the paracellular pathway of intestinal epithelial cells and maintaining the functional integrity of IMB. Their levels reflect the intestinal barrier function (Chen et al., 2022). Mice in the DSS-induced MC group showed notably lower levels of Occludin, Claudin-1, and ZO-1 protein expression in their colons compared to the NC group (*p* < 0.0001). The synbiotic group’s levels of Occludin, Claudin-1, and ZO-1 were considerably higher (*p* < 0.05) than those of the MC group, whereas the M-H group’s expression levels of Occludin and Claudin-1 were comparable to those of the NC group (Figure 5).

According to the findings, mixed probiotics combined with oligofructose may help preserve intestinal barrier integrity by boosting TJ protein expression and lowering LPS, DAO, and D-lactic levels.

### 3.5 Effect of synbiotics on intestinal metagenomic profile in colitis mice

**FIGURE 6:**
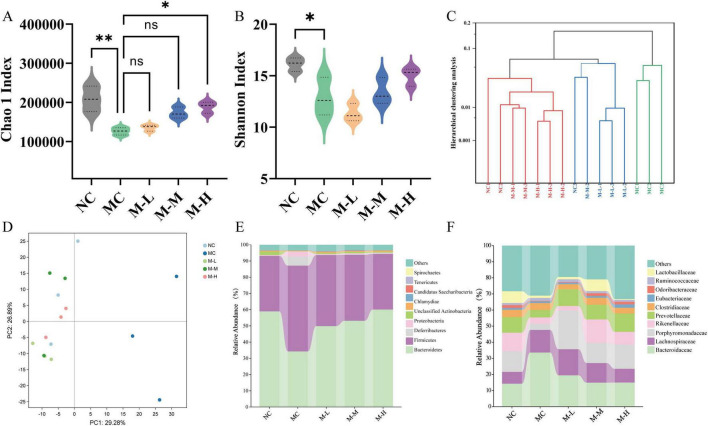
Effect of synbiotics on intestinal metagenomic profile in colitis mice. **(A)** Chao 1 Index. **(B)** Shannon Index. **(C)** Hierarchical clustering analysis. **(D)** PCA analysis. **(E)** Relative abundances of the gut microbiota at the phylum level. **(F)** Relative abundances of the gut microbiota at the family level (*p < 0.05, **p < 0.01, ***p < 0.001, ****p < 0.0001 and “ns” indicates non-significant, data represent mean ± SE).

Fecal metagenomic sequencing was used to monitor the effect of post-biological use on the fecal intestinal microbiota of rats with colitis. The α-diversity index was mainly evaluated by Chao1 index and Shannon index. Mice with DSS-induced colitis had less diversity and richness of intestinal microbes, as evidenced by the MC group’s considerably lower Chao1 and Shannon indices as compared to the NC group (*p* < 0.01 and *p* < 0.05, respectively) (Figures 6A,B). At the same time, the M-H group remarkedly increased the Chao1 index and improved the diversity of intestinal microorganisms (*p* < 0.05). Simultaneously, the main coordinate analysis was used to assess the impact of varying dosages of mixed probiotics combined with oligofructose on the intestinal microbiota structure of mice (Bray-Curtis distance; Figure 6C) was used to compare the inter-group differences in Bray-Curtis distance. This distance evaluates the similarity of fecal microbial communities between the two groups of rats; the smaller the distance, the higher the similarity between the groups. The findings demonstrated that mices with DSS-induced colitis had a substantially different intestinal microbiota structure than rats in the control group that did not receive DSS therapy. Notably, the Bray-Curtis gap between the M-L group and the NC group was substantially larger than the Bray-Curtis distance between the M-M group, the M-H group, and the NC group. The above results suggest that administration of high or medium doses of mixed probiotics combined with oligofructose may have a stronger regulatory effect on the intestinal microbiota of rats with colitis. At the same time, principal component analysis (PCA) showed the same results. The intestinal microorganisms of the mixed probiotics combined with oligofructose treatment group and the NC group were more similar, and the intestinal microorganisms of the MC group were signally changed (Figure 6D). In all groups, intestinal Bacteroidetes and Firmicutes accounted for more than 90% of the total intestinal microbiota. In all groups, Firmicutes and Bacteroidetes accounted for more than 80% of the total intestinal microbiota (Figure 6E). Compared with the NC group, the MC group had fewer Bacteroidetes and more Firmicutes, but the mixed probiotics combined with oligofructose partially reversed this unfavorable probiotic trend. At the family level, the intestinal microbiota of mice in each group was dominated by *Bacteroidaceae*, *Lachnospiraceae*, *Porphyromonadaceae*, and *Rikenellaceae*. Compared with the normal group, the intestinal microbiota of the model group mice *Bacteroidaceae* and *Lachnospiraceae* increased, and *Porphyromonadaceae* and *Rikenellaceae* decreased (Figure 6F).

### 3.6 Restoration effect of synbiotics on DSS-induced intestinal microbiota characteristic disturbance

**FIGURE 7:**
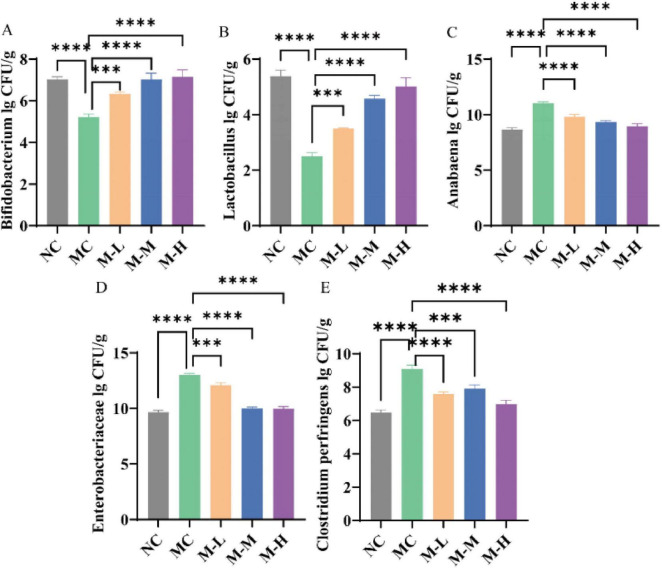
Restoration effect of synbiotics on DSS-induced intestinal microbiota characteristic disturbance. **(A)** *Bifidobacterium.* **(B)** *Lactobacillus.* **(C)** *Anabaena.* **(D)** *Enterobacteriaceae.* **(E)** *Clostridium perfringens* (*p < 0.05, **p < 0.01, ***p < 0.001, ****p < 0.0001 and “ns” indicates non-significant, data represent mean ± SE).

As shown in Figure 7, DSS changed the mice’s intestinal microbiota composition and had different levels of impact with different microbial flora when compared to NC group. The *Enterococcus*, *Enterobacter*, and *Clostridium perfringens* in the feces of the mice in the MC group increased significantly (*p* < 0.0001), while *Lactobacillus* and *Bifidobacterium* decreased markedly (*p* < 0.0001). Compared with the MC group, the content of *Bifidobacterium* and *Lactobacillus* in the fecal flora of mice fed with synbiotics increased outstandingly (*p* < 0.001), and the content of *Enterococcus*, *Enterobacter* and *Clostridium perfringens* decreased remarkedly (*p* < 0.001). Synbiotics increased the content of beneficial bacteria and restored some bacterial species to normal flora content. It had a significant repair effect on intestinal microbiota imbalance and maintained the balance of intestinal microbiota.

### 3.7 Regulation effect of synbiotics on DSS-induced intestinal function of gut microbiota

**FIGURE 8:**
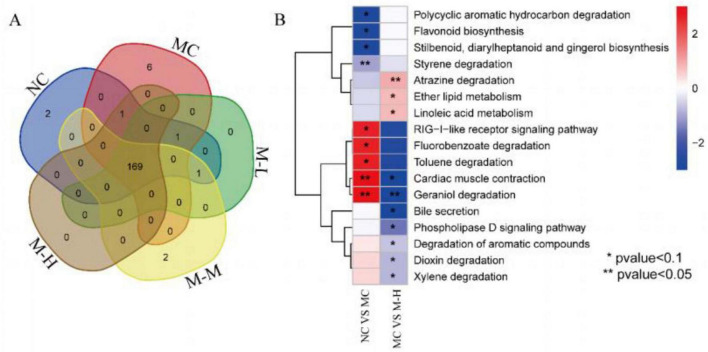
Regulation effect of synbiotics on DSS-induced intestinal function of gut microbiota. **(A)** Venn diagram showing the distribution of identified metabolic pathways in different groups. **(B)** Heatmap showing the relative abundance of the identified differential pathways between NC vs. MC and MC vs. M-H.

The functional pathways encoded by the fecal microorganisms of the five groups of mice were analyzed. A total of 182 metabolic pathways were identified, of which 169 metabolic pathways were found in the five groups, of which NC, MC and M-M had 2, 6, and 2 specific metabolic pathways respectively (Figure 8A). Compared with the NC group, the metabolic pathways significantly upregulated in the MC group were: RIG-I-like receptor signaling pathway, Fluorobenzoate degradation, Toluene degradation, Cardiac muscle contraction, Geraniol degradation, and the metabolic pathways notably decreased were: Polycyclic aromatic hydrocarbon degradation, Flavonoid biosynthesis, Stilbenoid, diarylheptanoid, and qingerol biosynthesis; compared with the MC group, the metabolic pathways significantly upregulated in the M-H group were: Atrazine degradation, Ether lipid metabolism, Linoleic acid metabolism, and the metabolic pathways outstandingly decreased were: Cardiac muscle contraction, Geraniol degradation, Bile secretion, Phospholipase D signaling pathway, Degradation of aromatic compounds, Dioxin degradation, Xylene degradation (Figure 8B).

### 3.8 Effects of synbiotics on the contents of short-chain fatty acids in colitis mice

About 90% of short chain fatty acids (SCFAs) are organic fatty acids made up of 1–6 carbon atoms, with acetate, propionate, and butyrate being the most prevalent. SCFAs have the ability to directly improve intestinal barrier function, preserve the integrity of the epithelial barrier, stimulate cell proliferation and differentiation, and modulate mucosal immunity (Liu et al., 2021). Acetate, propionate, and butyrate-the three primary SCFAs identified in mouse feces-were qualitatively and quantitatively analyzed in this work using gas chromatography-mass spectrometry.

**FIGURE 9:**
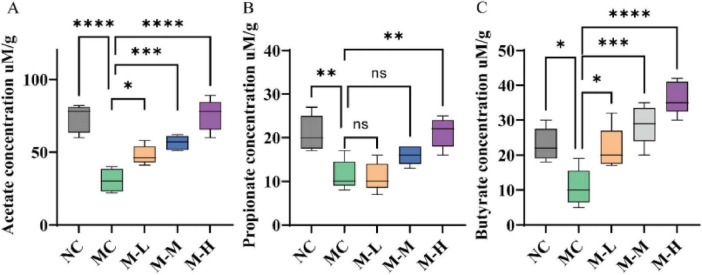
Effect of synbiotics on short-chain fatty acids in colitis mice. Content of **(A)** Acetate, **(B)** Propionate, and **(C)** Butyrate in the colon (*p < 0.05, **p < 0.01, ***p < 0.001, ****p < 0.0001 and “ns” indicates non-significant, data represent mean ± SE).

As illustrated in Figure 9, the acetate level of mice in the MC group decreased from 73.40 μM/g to 30.60 μM/g, the propionate level decreased from 21.00 μM/g to 11.40 μM/g, and the butyrate level decreased from 23.00 μM/g to 10.80 μM/g, which were significantly different from those in the control group (*p* < 0.0001, *p* < 0.01, and *p* < 0.05, respectively). The concentration of SCFAs was enhanced by the mixed probiotics and oligofructose as compared to the MC group, and the M-H group had a more significant intervention effect on acetic acid, propionic acid, and butyric acid (*p* < 0.0001, *p* < 0.01, and *p* < 0.0001, respectively). This situation should be because the mixed probiotics combined with oligofructose should have stimulated the growth of certain bacteria that generate SCFAs in the colon.

## 4 Discussion

There is a complex link between intestinal microbial imbalance and IBD, a chronic intestinal disease (Ng et al., 2017). Probiotics, prebiotics, and their combination (synbiotics) have been shown to enhance gut microbiota and may be used as therapies for IBD (Cai et al., 2021; Kim et al., 2021; Martyniak et al., 2021). Few research, however, have looked at the synergistic mechanisms of probiotics and prebiotics as well as the regulatory effects of synbiotics on the disruption of intestinal microbiota in mice with DSS-induced colitis.

In this study, we found that mixed probiotics-oligofructose synbiotics can effectively restore colitis, such as the recovery of body weight and intestinal tissue structure, the reduction of inflammatory cytokine expression, the increase of tight junction protein expression, etc., regulate the intestinal microbial structure and promote the production of SCFAs, among which high-dose mixed probiotics-oligofructose synbiotics are most effective. The above results show that mixed probiotics and oligofructose have synergistic effects and are dose-dependent for mixed probiotics, which is conducive to guiding the further development and utilization of synbiotics composed of probiotics and prebiotics.

Patients with IBD and colitis models present with inflammatory responses accompanied by colonic damage, and regulating host cytokine levels is a key way to alleviate intestinal inflammation (Brudek, 2019; Nguyen et al., 2021). IL-1β, IL-6, and IL-10 are important pro-inflammatory cytokines and anti-inflammatory factors (Al-Sadi et al., 2008; Al-Sadi et al., 2010). Patients with IBD and colitis models present with inflammatory responses accompanied by colonic damage, and regulating host cytokine levels is a key way to alleviate intestinal inflammation (Kühn et al., 1993; Wu et al., 2019). IL-10, an anti-inflammatory cytokine mostly generated by Treg cells, differs from the pro-inflammatory cytokines IL-1β and IL-6. It is essential for protecting against IBD because it inhibits the immune response to infections, hence reducing host damage (Wu et al., 2019). According to the study’s findings, synbiotics can increase the expression of the anti-inflammatory factor IL-10 and decrease the expression of pro-inflammatory factors (IL-1β and IL-6) when compared to the DSS group. This may be the main way of reducing intestinal inflammation and damage brought on by DSS.

The expression of TJ proteins is widely considered to be the main indicator for assessing the severity of intestinal damage. Transmembrane proteins such as claudin-1, occluding, and ZO-1 are key components that connect epithelial cells, regulate epithelial polarity and control the movement of solutes and fluids carrying bacteria in the intercellular matrix (Chen et al., 2021). The results showed that synbiotics can promote the expression of ZO-1, claudin-1, and occludin, and play a protective role in maintaining the integrity of the intestinal barrier.

Additionally, synbiotics remarkedly lowered the intestinal microbiota disorder symptoms in colitis-affected mice. Alpha and beta diversity analysis revealed that the treatment group exhibited some improvement, but the diversity and composition structure of the intestinal microbiota in the control group were obviously different from those in the DSS group. The MC group’s composition distribution at the taxonomic level of flora showed a significant increase in *Bifidobacterium* and *Lactobacillus* content, a decrease in Bacteroidetes, and an increase in Firmicutes. Functional annotation analysis of metagenomic data was performed based on the Kyoto Encyclopedia of Genes and Genomes (KEGG) database. It is worth noting that synbiotics can regulate linoleic acid metabolism. The increased abundance of lactobacilli in the intestine can promote linoleic acid metabolism and produce metabolites such as conjugated linoleic acid (CLA), which has multiple biological functions such as anti-inflammatory and anti-tumor, and effectively regulates the production of inflammatory cytokines (Su et al., 2024). In addition, research by Liu Yi and others has shown that the accumulation of linoleic acid can aggravate the occurrence and development of colitis (Jia et al., 2024).

In addition to the predicted linoleic acid derivatives, SCFAs are also important metabolites of prebiotics, which have the function of regulating intestinal flora and strengthening the intestinal barrier. Oligofructose is usually not digested by the gastrointestinal tract, and most of it is broken down by intestinal microorganisms in the colon (*Bifidobacteria* are the main consumers), producing metabolites including SCFAs (Holmberg et al., 2024). Therefore, LC-MS was used to detect the content of SCFAs. The results showed that the content of SCFAs in the M-H group of mice increased significantly, and the levels of acetic acid and propionic acid were comparable to those in the control group. Mixed probiotics regulate intestinal flora by decomposing oligofructose and promoting the synthesis of SCFAs (Ni et al., 2021). The results mentioned above reveal the regulation of intestinal microbiota, the ability to reduce intestinal inflammation, and the combined effect of probiotics combined with prebiotics.

## 5 Conclusion

The study showed that the synbiotic combination of mixed probiotics and oligofructose effectively alleviated DSS-induced colitis by alleviating clinical symptoms, regulating inflammatory cytokines, and enhancing intestinal barrier function. The combination of high-dose mixed probiotics and oligofructose had the best effect, which was dose-dependent. In addition, synbiotic treatment played a positive role in regulating the composition and structure of intestinal microorganisms, especially by enriching SCFA-producing bacteria, such as *Bifidobacterium* and *Lactobacillus*, and promoting the increase of protective SCFAs levels, including butyrate, which activated anti-inflammatory and barrier-enhancing pathways to alleviate colonic damage. This study demonstrated that the effect of the synbiotic combination of mixed probiotics and oligofructose on relieving colitis symptoms depends on the dose of mixed probiotics, which is conducive to guiding the further development and use of synbiotic products of probiotics and oligofructose; it clarified the mechanism of action of the synbiotic combination of mixed probiotics and oligofructose to regulate linoleic acid metabolism and SCFAs metabolism by reorganizing the structure and composition of intestinal microbiota to alleviate DSS-induced colitis, providing new insights into the mechanism of action of synbiotics in alleviating UC.

## Data Availability

The original contributions presented in the study are publicly available. This data can be found here: https://www.ncbi.nlm.nih.gov/, PRJNA1289890.
